# Testing the construct validity of real-world digital mobility outcomes in multiple sclerosis

**DOI:** 10.1177/13524585261464537

**Published:** 2026-08-02

**Authors:** Gavin Brittain, Michael Long, Ellen Buckley, Clemens Becker, Joren Buekers, Martí de las Heras, Claudia Armengol, Sarah Koch, Julia Braun, Brian Caufield, Giancarlo Comi, Gloria Dalla Costa, Anja Frei, Heiko Gassner, Anisoara Ionescu, Letizia Leocani, Lynn Rochester, Veit Rothhammer, David Singleton, Klarissa Hanja Stuerner, David Paling, Krishnan Padmakumari Sivaraman Nair, Arshad Majid, Giampaolo Brichetto, Beatrix Vereijken, Vita Lanfranchi, Carl-Philipp Jansen, Lou Sutcliffe, Silvia Del Din, Isabel Neatrour, Andrea Cereatti, Walter Maetzler, Clint Hansen, Daniel Rooks, Basil Sharrack, Judith Garcia-Aymerich

**Affiliations:** Sheffield Institute for Translational Neuroscience (SITraN), School of Medicine and Population Health, The University of Sheffield, Sheffield, UK; NIHR Sheffield Biomedical Research Centre, Sheffield Teaching Hospital NHS Foundation Trust, Sheffield, UK; School of Computer Science, The University of Sheffield, Sheffield, UK; NIHR Sheffield Biomedical Research Centre, Sheffield Teaching Hospital NHS Foundation Trust, Sheffield, UK; School of Computer Science, The University of Sheffield, Sheffield, UK; School of Medicine and Population Health, INSIGNEO Institute for in Silico Medicine, The University of Sheffield, Sheffield, UK; Robert Bosch Gesellschaft für Medizinische Forschung, Stuttgart, Germany; ISGlobal, Barcelona, Spain; Universitat Pompeu Fabra (UPF), Barcelona, Spain; CIBER Epidemiología y Salud Pública (CIBERESP), Barcelona, Spain; ISGlobal, Barcelona, Spain; Universitat Pompeu Fabra (UPF), Barcelona, Spain; CIBER Epidemiología y Salud Pública (CIBERESP), Barcelona, Spain; ISGlobal, Barcelona, Spain; Universitat Pompeu Fabra (UPF), Barcelona, Spain; CIBER Epidemiología y Salud Pública (CIBERESP), Barcelona, Spain; ISGlobal, Barcelona, Spain; CIBER Epidemiología y Salud Pública (CIBERESP), Barcelona, Spain; Department of Sport, Exercise and Health, University of Basel, Basel, Switzerland; Epidemiology, Biostatistics and Prevention Institute, University of Zurich, Zurich, Switzerland; Insight – Centre for Data Analytics, University College Dublin, Dublin, Ireland; UCD School of Public Health, Physiotherapy and Sports Science, University College Dublin, Dublin, Ireland; Department of Neurorehabilitation Sciences, Casa di Cura IGEA, Milano, Italy; Universita San Raffaele, Milan, Italy; Harvard T.H. Chan School of Public Health, Harvard University, Boston, MA, USA; Epidemiology, Biostatistics and Prevention Institute, University of Zurich, Zurich, Switzerland; Department of Molecular Neurology, University Hospital Erlangen, Erlangen, Germany; Fraunhofer Institute for Integrated Circuits IIS, Erlangen, Germany; EPFL, Lausanne, Switzerland; San Raffaele Scientific Institute, Vita-Salute San Raffaele University, Milano, Italy; Translational and Clinical Research Institute, Faculty of Medical Sciences, Newcastle University, Newcastle upon Tyne, UK; Newcastle Biomedical Research Centre (BRC), National Institute for Health and Care Research (NIHR), Newcastle University and The Newcastle upon Tyne Hospitals NHS Foundation Trust, Newcastle upon Tyne, UK; Department of Neurology, University Hospital Erlangen, Erlangen, Germany; Insight – Centre for Data Analytics, University College Dublin, Dublin, Ireland; UCD School of Public Health, Physiotherapy and Sports Science, University College Dublin, Dublin, Ireland; Department of Neurology, University Hospital Schleswig-Holstein, Kiel, Germany; NIHR Sheffield Biomedical Research Centre, Sheffield Teaching Hospital NHS Foundation Trust, Sheffield, UK; Sheffield Institute for Translational Neuroscience (SITraN), School of Medicine and Population Health, The University of Sheffield, Sheffield, UK; NIHR Sheffield Biomedical Research Centre, Sheffield Teaching Hospital NHS Foundation Trust, Sheffield, UK; Sheffield Institute for Translational Neuroscience (SITraN), School of Medicine and Population Health, The University of Sheffield, Sheffield, UK; NIHR Sheffield Biomedical Research Centre, Sheffield Teaching Hospital NHS Foundation Trust, Sheffield, UK; AISM Rehabilitation Service of Liguria, Genoa, Italy; Department of Neuromedicine and Movement Science, Norwegian University of Science and Technology (NTNU), Trondheim, Norway; School of Computer Science, The University of Sheffield, Sheffield, UK; Geriatric Center, Medical Faculty Heidelberg, Heidelberg University, Heidelberg, Germany; Translational and Clinical Research Institute, Faculty of Medical Sciences, Newcastle University, Newcastle upon Tyne, UK; Newcastle Biomedical Research Centre (BRC), National Institute for Health and Care Research (NIHR), Newcastle University and The Newcastle upon Tyne Hospitals NHS Foundation Trust, Newcastle upon Tyne, UK; Translational and Clinical Research Institute, Faculty of Medical Sciences, Newcastle University, Newcastle upon Tyne, UK; Newcastle Biomedical Research Centre (BRC), National Institute for Health and Care Research (NIHR), Newcastle University and The Newcastle upon Tyne Hospitals NHS Foundation Trust, Newcastle upon Tyne, UK; Translational and Clinical Research Institute, Faculty of Medical Sciences, Newcastle University, Newcastle upon Tyne, UK; Newcastle Biomedical Research Centre (BRC), National Institute for Health and Care Research (NIHR), Newcastle University and The Newcastle upon Tyne Hospitals NHS Foundation Trust, Newcastle upon Tyne, UK; Department of Electronics and Telecommunications, Polytechnic University of Turin, Turin, UK; Department of Neurology, University Hospital Schleswig-Holstein and Kiel University, Kiel, Germany; Department of Neurology, University Hospital Schleswig-Holstein and Kiel University, Kiel, Germany; Translational Medicine, Biomedical Research, Novartis AG, Cambridge, MA, USA; Sheffield Institute for Translational Neuroscience (SITraN), School of Medicine and Population Health, The University of Sheffield, Sheffield, UK; NIHR Sheffield Biomedical Research Centre, Sheffield Teaching Hospital NHS Foundation Trust, Sheffield, UK; ISGlobal, Barcelona, Spain; Universitat Pompeu Fabra (UPF), Barcelona, Spain; CIBER Epidemiología y Salud Pública (CIBERESP), Barcelona, Spain

**Keywords:** Digital mobility outcomes, wearable devices, multiple sclerosis, construct validity, digital health

## Abstract

**Background:**

Wearable devices can measure real-world mobility and generate metrics known as digital mobility outcomes (DMOs). For people with multiple sclerosis (pwMS), these DMOs have not been clinically validated. This study aimed to evaluate the construct (convergent, divergent, and known groups) validity of a comprehensive panel of 24 DMOs in pwMS.

**Methods:**

PwMS were recruited to the Mobilise-D Clinical Validation Study, which involved 7-days of real-world mobility monitoring with a lower-back-worn wearable device. DMOs of walking amount, pattern, pace, rhythm, and variability domains were assessed. Convergent validity was evaluated against established clinical constructs (Expanded Disability Status Scale (EDSS), Timed 25-Foot Walk, Multiple sclerosis (MS) Walking Scale-12, and Patient-Determined Disease Steps) using a priori hypotheses. Divergent validity was tested against systolic blood pressure. Known-groups validity was assessed across three EDSS strata. An expert panel completed a standardised consensus process.

**Results:**

We included 556 participants: 65% women, mean (SD) age of 52.3 (10.7) years, and median (p25–p75) EDSS 5 (4–6). Convergent, divergent, and known-groups validity were supported for 19, 24, and 23 DMOs, respectively. Nineteen DMOs, covering all domains, reached consensus agreement.

**Conclusion:**

This study confirms the construct validity of 19 DMOs across all walking domains in pwMS, supporting their use in clinical research and practice. www.isrctn.com/ISRCTN12051706.

## Introduction

Multiple sclerosis (MS) is a chronic neuroinflammatory and neurodegenerative disorder that affects 2.9 million people worldwide.^1^ It results in mobility impairment in approximately 90% of affected people during their lifetime,^2^ with 76% requiring a walking aid during their disease course.^3^ Declining mobility is the most important perceived disability to patients^4^ and has significant functional consequences as it correlates strongly with unemployment,^5,6^ loss of income,^7^ rising healthcare costs,^8^ and reduced quality of life.^2^

The most commonly used clinical assessment tools in MS, such as the Kurtzke Expanded Disability Status Scale (EDSS)^9,10^ or the Timed 25-Foot Walk test (T25FW),^11^ assess predominantly how far or how fast people with multiple sclerosis (pwMS) can walk. These measures evaluate mobility capacity (the ‘best’ a patient can do under direct observation). Mobility perception (i.e. a person’s feeling of their mobility) is often assessed by using patient-reported outcome measures such as the 12-item Multiple Sclerosis Walking Scale (MSWS-12)^12^ or the patient-determined disease step score (PDDS).^13,14^ However, the assessment of mobility performance (i.e. what a person actually does in daily-life conditions) is much less established in MS,^15^ despite the growing evidence that various laboratory-collected gait parameters are sensitive to disease progression in patients with stable EDSS scores.^16,17^ Real-world mobility monitoring captures mobility performance, reflecting how individuals actually move during daily life. This distinction is important because real-world mobility is influenced by behavioural, environmental, and disease-related factors such as fatigue, symptom fluctuations, and activity patterns, which may not be captured during brief supervised clinical assessments.

There is growing evidence of the importance of digital technology in clinical assessment,^18^ and more specifically, in mobility performance assessment, of clinical populations.^15,19–21^ Wearable devices may be used by pwMS to track and analyse mobility performance and to provide sensitive assessments of mobility disability, earlier prediction of subclinical disease progression, and overall health status. This may enable accurate monitoring of progression independent of relapse activity and could be a useful outcome measure in treatment trials and clinical care. Recently, the Mobilise-D technical validation study developed an open-source data processing pipeline that enables the assessment of mobility performance using a body worn device in diverse clinical populations, including MS. The Mobilise-D pipeline enables the evaluation of various digital mobility outcomes (DMOs) to describe a person’s mobility performance in terms of walking activity, measuring both amount (e.g. step count) and pattern (e.g. number of walking bouts) of gait, as well as measuring pace (e.g. gait speed), rhythm (e.g. cadence), and bout-to-bout variability.^15^ When tested against gold standard methods for measurement of walking gait, such as motion capture systems and instrumented walkways, these DMOs exhibited good to excellent criterion validity in MS.^22–25^ These studies confirm that wearable sensors can accurately quantify spatiotemporal gait parameters under controlled conditions. However, criterion validity alone does not establish clinical utility. For clinical application, it is necessary to demonstrate that these measures meaningfully reflect disease severity, functional status, and clinically relevant constructs when assessed in real-world settings. Therefore, further validation examining the clinical relevance of DMOs is required.

The objective of this study is to examine the construct validity of real-world walking activity and gait DMOs in pwMS using convergent, divergent, and known-groups validity.

## Methods

### Design and participants

This is a cross-sectional study using baseline data from the Mobilise-D Clinical Validation Study (CVS; ISRCTN number: 12051706), a multicentre observational cohort study, aiming to clinically validate novel DMOs in four clinical conditions (chronic obstructive pulmonary disease, Parkinson’s disease, MS, and proximal femur fracture),^26^ with follow-up for up to 2 years at 6-month intervals (a total of five visits).

Participants with MS were recruited from April 2021 to September 2022 in four cities across three European countries: Sheffield, United Kingdom; Milan, Italy; and Kiel and Erlangen, Germany. Ethical approval was obtained in accordance with local policy, and informed written consent was obtained. People with a diagnosis of MS based on the revised 2017 McDonald’s criteria,^27^ an EDSS score between 3.0 and 6.5, subjective or objective disability worsening over the previous 2 years and a willingness to wear a mobility monitor device for 7 days were eligible for inclusion. Participants were excluded if they had experienced a clinical relapse within the previous 30 days or a new significant illness, predefined elsewhere,^26^ within the previous 3 months.

This study considered only baseline data for pwMS from the locked Mobilise-D CVS database version 7.2.

### Digital mobility assessment

DMOs were measured using one of two metrologically equivalent single wearable devices: the MoveMonitor + device (McRoberts B.V., The Hauge, The Netherlands), worn on a belt around the waist positioned at the centre of the lower back, or the Axivity AX6 (Axivity Ltd., Newcastle Upon Tyne, UK), affixed to the participants’ skin at the centre of the lower back using an adhesive patch, continuously for 7 days, removing only for bathing, swimming or medical investigations, such as magnetic resonance scans. The devices are considered technically comparable, with an excellent agreement between devices (intraclass correlation coefficient (ICC) ⩾0.987) having been found during technical validation in both laboratory and free-living conditions, indicating that differences between devices were smaller than the expected measurement error associated with walking speed estimation using a single wearable device. The devices consisted of an inertial measurement unit (IMU) integrating a triaxial accelerometer (range of ±8 g) and a triaxial gyroscope (range of ±2000 deg/s), with a sampling frequency of 100 Hz. Standardised sensor data were processed using the validated Mobilise-D processing pipeline, and gait features were extracted and walking bout (WB)-level DMOs were obtained, as previously described.^22,25,28^ A WB was defined as a continuous walking sequence comprising at least two successive strides involving both feet (i.e. a minimum of four steps).^29^ The minimum wear time required for a valid and reliable measurement was >12 hours during waking hours (07:00–22:00 hours) for at least 3 days (no weekday/weekend restrictions).^30^ Participants who did not meet the minimum threshold were excluded from subsequent analyses. According to the protocol outlined previously, WB-level DMOs were aggregated to provide 24 weekly DMOs (see Supplemental Table S1).^31^ Twenty-four DMOs encompassing walking activity (amount and pattern) and gait domains (pace, rhythm, and bout-to-bout variability) were obtained (see Table 2 for full list of DMOs).

### Other measures

Demographic characteristics and general descriptive measures were collected, including age, gender, disease duration, MS subtype, comorbidities, mobility aid use, and self-reported falls history. Clinical and patient-reported outcome measures were administered according to standardised protocols by suitably trained researchers during in-person study visits. These included the EDSS, T25FW, MSWS-12, and PDDS. A single seated resting blood pressure was measured with an automated sphygmomanometer after at least 5 minutes of rest. All assessments were performed immediately before the period of digital mobility monitoring.

### Statistical analysis

Statistical analysis was planned a priori and conducted using R (v4.4.0). Sample size requirements were determined based on Fisher’s z-test for correlations, assuming an alpha of 0.05 and 80% power. To accommodate the varying expected correlations across the 24 DMOs under investigation, sample size was calculated for three distinct correlation thresholds: coefficients of 0.6 required 19 participants; coefficients of 0.4 required 47 participants; and coefficients of 0.2 required 194 participants. The final recruitment target was set to satisfy the most conservative threshold (0.2) and to maintain statistical integrity during planned subgroup analyses. The analysis used a pairwise complete-case approach including only observations where both the DMO and the corresponding construct measure were available. Cohort characteristics and DMOs were described using mean and SD, or median and p25–p75, based on normality.

To test construct validity, we followed the COnsensus-based Standards for the selection of health Measurement INstruments (﻿COSMIN) framework (www.cosmin.nl) by formulating a priori hypotheses regarding the expected direction and magnitude of correlations between each DMO and its respective clinical anchors. To assess the convergent validity of the 24 DMOs with related constructs (EDSS, T25FW, MSWS-12, and PDDS), Pearson (*r*) or Spearman (*ρ*) coefficients were used, depending on the variable distribution. To determine whether a DMO supported convergent validity, we anticipated expected correlation coefficients from pilot study results,^22^ previous literature, and expert consultation (see Supplemental Table S2). Cohen’s^32^ guidelines of 0.1, 0.3, and 0.5 were used to classify correlation coefficients as weak, moderate, and strong, respectively. Subgroup analysis for convergent validity was conducted to examine whether the strength of the associations between DMOs and clinical constructs was consistent across the following participant strata: gender, age (by tertiles: <50, 50–57, and >57 years), height (tertiles: <165, 165–174, and >174 cm), weight (tertiles: <66, 66–88, and >88 kg), recruitment country (Germany, Italy, and the United Kingdom), and falls status in the previous year (⩾1 falls (faller) and 0 falls (non-faller)).

Divergent validity was assessed by testing the correlations of each DMO with systolic blood pressure value, a priori expecting correlation coefficients to reflect no relationship to any of the DMOs (|r/ρ|< 0.2). Resting blood pressure was included as a comparator for divergent validity because it reflects cardiovascular status but is not expected to be directly associated with real-world mobility performance. Systolic blood pressure was used as it is the most commonly reported blood pressure parameter and the primary measure used in cardiovascular risk assessment.

Known-groups validity was assessed by testing the distribution of the DMOs according to the groups defined by EDSS scores: 3.0–4.0 (*n* = 192), 4.5–5.5 (*n* = 120), and 6.0–6.5 (*n* = 241), reflecting the ability to ambulate in a single bout over 500 m unaided, between ⩾100 and 499 m unaided, and requiring walking aids, respectively. Known-groups validity was supported when ordinal consistency was demonstrated through a significant *p*-for-trend (*p* < 0.05) in linear regression models. Given the validation-focused nature of this study and the use of a priori hypotheses for each of the 24 DMOs, no formal correction for multiple comparisons was applied.^33^

Finally, construct validity was established through a formal expert consensus process involving a multidisciplinary panel of 11 experts from diverse backgrounds (neurology, neurorehabilitation, epidemiology, statistics, exercise physiology, data science, digital health, and gait biomechanics). No significant conflicts of interest were identified that precluded participation in the voting process. The consensus was conducted in two distinct phases. First, experts independently evaluated each DMO via an anonymised, blinded survey. Each DMO was assessed against the predefined validity assumptions described above, without experts having access to the responses of their peers. Following the individual assessments, a consensus meeting was held to review the results. Disagreements were handled through a structured discussion aimed at identifying the clinical or technical reasoning behind differing opinions. This ensured transparency and allowed for the reconciliation of divergent perspectives through evidence-based dialogue. A DMO was classified as having demonstrated construct validity if a majority of experts (>50%) supported it. To reflect the nuances of the panel’s deliberations and provide a transparent measure of the strength of evidence, the final distribution of votes and the discussion content for each DMO are reported.

## Results

From a total of 602 participants recruited across 18 months, reported in detail elsewhere,^34^ 556 participants were included for the current analysis (Table 1). There were no differences between included and excluded participants in any sociodemographic or clinical variables except T25FW (Supplemental Table S3). Of the 46 (8%) excluded participants, 30 (5%) had missing data, and 16 (2.7%) did not meet the minimum wear time. The included sample was predominantly women (64.8%), with a mean age of 52.3 years (SD = 10.7) and a median EDSS score of 5.0. During the digital mobility assessment, each participant walked a median of 5258 WB steps (p25–p75 = 2944–7987) daily, with a mean cadence of 88 steps per minute (*SD* = 6; Table 2).

**Table 1.** Demographic and clinical variables for all included pwMS and categorised by EDSS groups.

	Total^a^ *n* = 556	EDSS categories		
		3.0–4.0 *n* = 192 (35%)	4.5–5.5 *n* = 120 (22%)	6.0–6.5 *n* = 241 (43%)
Age, mean (SD)	52 (11)	49 (11)	52 (10)	55 (10)
Gender, woman, *n* (%)	360 (65%)	111 (58%)	85 (71%)	162 (67%)
Marital status, married, *n* (%)	375 (67%)	129 (67%)	82 (68%)	162 (67%)
Education, *n* (%)				
*College degree or higher*	190 (34%)	80 (42%)	34 (28%)	76 (32%)
*Tertiary education*	152 (27%)	42 (22%)	39 (32%)	71 (29%)
*High school*	143 (26%)	52 (27%)	31 (26%)	57 (24%)
*Less than high school (<10 years)/others*	71 (13%)	18 (9%)	16 (13%)	37 (15%)
Site, *n* (%)				
*Kiel*	44 (8%)	21 (11%)	4 (3%)	18 (7%)
*Erlangen*	14 (3%)	7 (4%)	2 (2%)	3 (1%)
*Sheffield*	280 (50%)	101 (53%)	63 (52%)	116 (48%)
*Milan*	218 (39%)	63 (33%)	51 (42%)	104 (43%)
Self-reported comorbidities, *n* (%)				
*Arthritis*	62 (11%)	19 (10%)	16 (13%)	27 (11%)
*Osteoporosis*	44 (8%)	9 (5%)	11 (9%)	24 (10%)
*Chronic heart failure*	7 (1%)	1 (1%)	0 (0%)	6 (2%)
*Diabetes*	20 (4%)	5 (3%)	5 (4%)	10 (4%)
*Depression*	121 (22%)	32 (17%)	31 (26%)	56 (23%)
*Anxiety*	75 (13%)	26 (14%)	21 (18%)	28 (12%)
*Visual impairment*	69 (12%)	21 (11%)	15 (12%)	33 (14%)
*Hearing impairment*	40 (7%)	7 (4%)	11 (9%)	22 (9%)
*Obesity*	124 (22%)	33 (17%)	31 (26%)	59 (24%)
MS type, *n* (%)				
*Primary progressive*	59 (11%)	17 (9%)	11 (9%)	31 (13%)
*Secondary progressive*	195 (35%)	23 (12%)	36 (30%)	135 (56%)
*Relapsing remitting*	300 (54%)	152 (79%)	71 (59%)	75 (31%)
MSWS-12, med (p25–p75)	41 (28–51)	26 (18–35)	41 (33–47)	50 (42–55)
T25FW (seconds), med (p25–p75)	8 (6–11)	6 (5–6)	8 (6–9)	12 (9–17)
PDDS (seconds), med (p25–p75)	3 (2–5)	1 (1–3)	3 (3–4)	5 (4–6)
Systolic blood pressure (mmHg), mean (SD)	130 (18)	129 (17)	131 (20)	132 (19)
Walking aids, *n* (%)				
Indoor	136 (24%)	2 (1%)	10 (8%)	124 (51%)
Outdoor	325 (58%)	32 (17%)	57 (48%)	236 (98%)
Falls in previous 12 months, median (p25–p75)	1 (0–3)	0 (0–2)	1 (0–3)	2 (0–4)
*0 falls*	234 (42%)	115 (60%)	52 (43%)	65 (27%)
*1 fall*	68 (12%)	26 (14%)	14 (12%)	28 (12%)
*⩾2 falls*	254 (46%)	51 (27%)	54 (45%)	148 (61%)

EDSS: Expanded Disability Status Scale; MS type: multiple sclerosis type; MSWS-12: 12-item Multiple Sclerosis Walking Scale; T25FW: Timed 25-Foot Walk; PDDS: Patient-Determined Disease Steps.

a Some constructs have missing values (NA): EDSS: 3 NA; T25FW: 13 NA; MSWS-12: 1 NA; PDDS: 1 NA; blood pressure systolic: 2 NA.

**Table 2.** Distribution and convergent and divergent validity (correlation, *r*, coefficients with corresponding 95% CIs) of DMOs in 556 people with MS and related constructs.

DMOs	DMO distribution	Convergent validity				Divergent validity
		EDSS	T25FW	MSWS-12	PDDS	Systolic blood pressure
**Walking activity**						
*Amount*						
Walking duration (min/day)	59 (33–87)	**−0.60 (−0.66, −0.54)**	**−0.54 (−0.61, −0.48)**	**−0.47 (−0.53, −0.39)**	**−0.58 (−0.64, −0.52)**	**0.03 (−0.05, 0.12)**
WB step count (steps/day)	5258 (2944–7987)	**−0.64 (−0.68, −0.58)**	**−0.58 (−0.64, −0.52)**	**−0.50 (−0.57, −0.43)**	**−0.62 (−0.67, −0.56)**	**0.02 (−0.06, 0.11)**
*Pattern*						
Number of WBs (*n*)	294 (158)	**−0.51 (−0.57, −0.44)**	**−0.45 (−0.53, −0.38)**	**−0.41 (−0.48, −0.33)**	**−0.51 (−0.56, −0.43)**	**0.07 (−0.01, 0.17)**
Number of WBs >10 seconds (*n*)	104 (61–145)	**−0.53 (−0.59, −0.46)**	**−0.46 (−0.53, −0.39)**	**−0.40 (−0.47, −0.33)**	**−0.51 (−0.57, −0.43)**	**0.08 (−0.01, 0.15)**
Number of WBs >30 seconds (*n*)	15 (7–25)	**−0.58 (−0.64, −0.52)**	**−0.52 (−0.59, −0.45)**	**−0.43 (−0.50, −0.36)**	**−0.54 (−0.59, −0.47)**	**0.02 (−0.07, 0.10)**
Number of WBs>60 seconds (*n*)	4 (1–8)	**−0.59 (−0.65, −0.53)**	**−0.55 (−0.61, −0.47)**	**−0.45 (−0.52, −0.38)**	**−0.55 (−0.62, −0.48)**	**−0.05 (−0.13, 0.04)**
WB duration (seconds)	7.7 (7.2–8.2)	**−0.22 (−0.30, −0.14)**	**−**0.13 (**−**0.22, **−**0.05)	**−**0.11 (**−**0.20, **−**0.03)	**−**0.17 (**−**0.24, **−**0.08)	**0.08 (−0.02, 0.16)**
P90 WB duration (seconds)	21.5 (18.3–25.6)	**−0.45 (−0.52, −0.38)**	**−0.39 (−0.47, −0.32)**	**−0.28 (−0.36, −0.21)**	**−0.38 (−0.46, −0.30)**	**−0.03 (−0.11, 0.05)**
WB duration bout-to-bout variability (%)	115 (86–153)	**−0.57 (−0.63, −0.51)**	**−0.59 (−0.64, −0.52)**	**−0.43 (−0.49, −0.36)**	**−0.52 (−0.58, −0.45)**	**−0.11 (−0.20, −0.03)**
**Gait**						
*Pace*						
Walking speed in shorter (10–30 seconds) WBs (m/s)	0.72 (0.10)	**−0.57 (−0.63, −0.51)**	**−0.59 (−0.65, −0.54)**	**−0.44 (−0.51, −0.37)**	**−0.57 (−0.63, −0.51)**	**−0.13 (−0.22, −0.05)**
Walking speed in longer (>30 seconds) WBs (m/s)	0.81 (0.17)	**−0.66 (−0.70, −0.60)**	**−0.68 (−0.73, −0.63)**	**−0.51 (−0.57, −0.43)**	**−0.65 (−0.71, −0.60)**	**−0.13 (−0.21, −0.05)**
P90 walking speed in WBs >10 seconds (m/s)	0.92 (0.17)	**−0.68 (−0.73, −0.64)**	**−0.71 (−0.74, −0.66)**	**−0.51 (−0.58, −0.44)**	**−0.68 (−0.72, −0.62)**	**−0.14 (−0.22, −0.05)**
P90 walking speed in longer (>30 seconds) WBs (m/s)	0.95 (0.23)	**−0.70 (−0.75, −0.66)**	**−0.73 (−0.77, −0.69)**	**−0.54 (−0.60, −0.47)**	**−0.70 (−0.74, −0.65)**	**−0.11 (−0.19, −0.03)**
Stride length in shorter (10–30 seconds) WBs (cm)	98 (11)	**−0.32 (−0.39, −0.24)**	**−0.35 (−0.43, −0.27)**	**−**0.19 (**−**0.27, **−**0.10)	**−0.28 (−0.36, −0.20)**	**−0.13 (−0.21, −0.04)**
Stride length in longer (>30 seconds) WBs (cm)	106 (15)	**−0.47 (−0.53, −0.39)**	**−0.50 (−0.56, −0.43)**	**−0.32 (−0.39, −0.24)**	**−0.42 (−0.49, −0.35)**	**−0.12 (−0.21, −0.02)**
*Rhythm*						
Cadence in all WBs (steps/min)	88 (6)	**−0.49 (−0.55, −0.42)**	**−0.47 (−0.53, −0.40)**	**−0.45 (−0.51, −0.37)**	**−0.54 (−0.60, −0.48)**	**−0.04 (−0.13, 0.04)**
Cadence in longer (>30 seconds) WBs (step/min)	90 (9)	**−0.60 (−0.65, −0.55)**	**−0.58 (−0.64, −0.53)**	**−0.51 (−0.57, −0.44)**	**−0.65 (−0.69, −0.59)**	**−0.08 (−0.16, 0.01)**
P90 cadence in longer (>30 seconds) WBs (steps/min)	97 (12)	**−0.66 (−0.70, −0.60)**	**−0.63 (−0.67, −0.57)**	**−0.55 (−0.62, −0.49)**	**−0.69 (−0.73, −0.63)**	**−0.08 (−0.16, 0.00)**
Stride duration in all WBs (seconds)	1.27 (0.08)	**0.30 (0.22, 0.37)**	**0.33 (0.24, 0.40)**	0.27 (0.20, 0.36)	0.31 (0.24, 0.39)	**−0.04 (−0.13, 0.05)**
Stride duration in longer (>30 seconds) WBs (seconds)	1.25 (0.13)	**0.46 (0.39, 0.53)**	**0.47 (0.40, 0.55)**	0.39 (0.31, 0.47)	**0.48 (0.40, 0.55)**	**0.08 (−0.00, 0.16)**
*Bout-to-bout variability*						
Walking speed bout-to-bout variability in longer (>30 seconds) WBs (%)	17 (6)	**−0.42 (−0.49, −0.34)**	**−0.45 (−0.52, −0.37)**	**−**0.31 (**−**0.38, **−**0.22)	**−0.43 (−0.49, −0.36)**	**−0.04 (−0.12, 0.05)**
Stride length bout-to-bout variability in longer (>30 seconds) WBs (%)	13 (5)	**−0.21 (−0.30, −0.13)**	**−0.22 (−0.30, −0.13)**	**−**0.16 (**−**0.25, **−**0.08)	**−**0.20 (**−**0.29, **−**0.11)	**0.00 (−0.08, 0.09)**
Cadence bout-to-bout variability (%)	12 (1)	0.04 (**−**0.05, 0.13)	0.06 (**−**0.04, 0.13)	**−**0.02 (**−**0.11, 0.06)	0.06 (**−**0.02, 0.14)	**−0.06 (−0.15, 0.02)**
Stride duration bout-to-bout variability (%)	18 (3)	0.24 (0.15, 0.31)	0.22 (0.12, 0.29)	0.23 (0.14, 0.30)	0.26 (0.17, 0.33)	**0.01 (−0.06, 0.10)**

Convergent validity is supported if the a priori correlation coefficients are met or exceeded (*r* >|expected correlation|) up to 0.9. Divergent validity is supported if|*r*| < 0.2. Values that meet the criteria are marked in bold in the results. Coefficients in bold represent those for which convergent or divergent validity is suggested.

### Convergent validity

Walking amount DMOs established moderate to strong correlations with clinical outcomes, consistent with the anticipated coefficients (ρ =|0.47|–|0.64|; Table 2). Pattern DMOs provided moderate to strong associations with clinical outcomes consistent with the expected coefficients (ρ =|0.28|–|0.59|), except for WB duration, which produced weak coefficients (ρ =|0.11|–|0.22|), thus lower than expected. Pace DMOs established moderate to strong coefficients matching the expected coefficients (ρ =|0.28|–|0.73|), except for the correlation between stride length in shorter bouts and MSWS-12 (ρ = **−**0.19). Rhythm DMOs demonstrated moderate to strong coefficients matching the expected coefficients (ρ =|0.30|–|0.69|), except for the correlation between stride duration in all bouts and MSWS-12 (ρ = 0.27) or PDDS (ρ = 0.31), and between stride duration in longer bouts and MSWS-12 (ρ = 0.39). For bout-to-bout variability DMOs, correlations demonstrated mild to moderate coefficients with clinical scores (ρ =|0.21|–|0.43|), except for stride duration variability, which produced weak to small correlations, and cadence variability, which produced no correlations with clinical outcomes.

Correlation coefficients were consistent across gender, age, height, weight, site, and falls status (see Figures 1 [Fig fig2-13524585261464537][Fig fig3-13524585261464537]–4).

**Figure 1.**
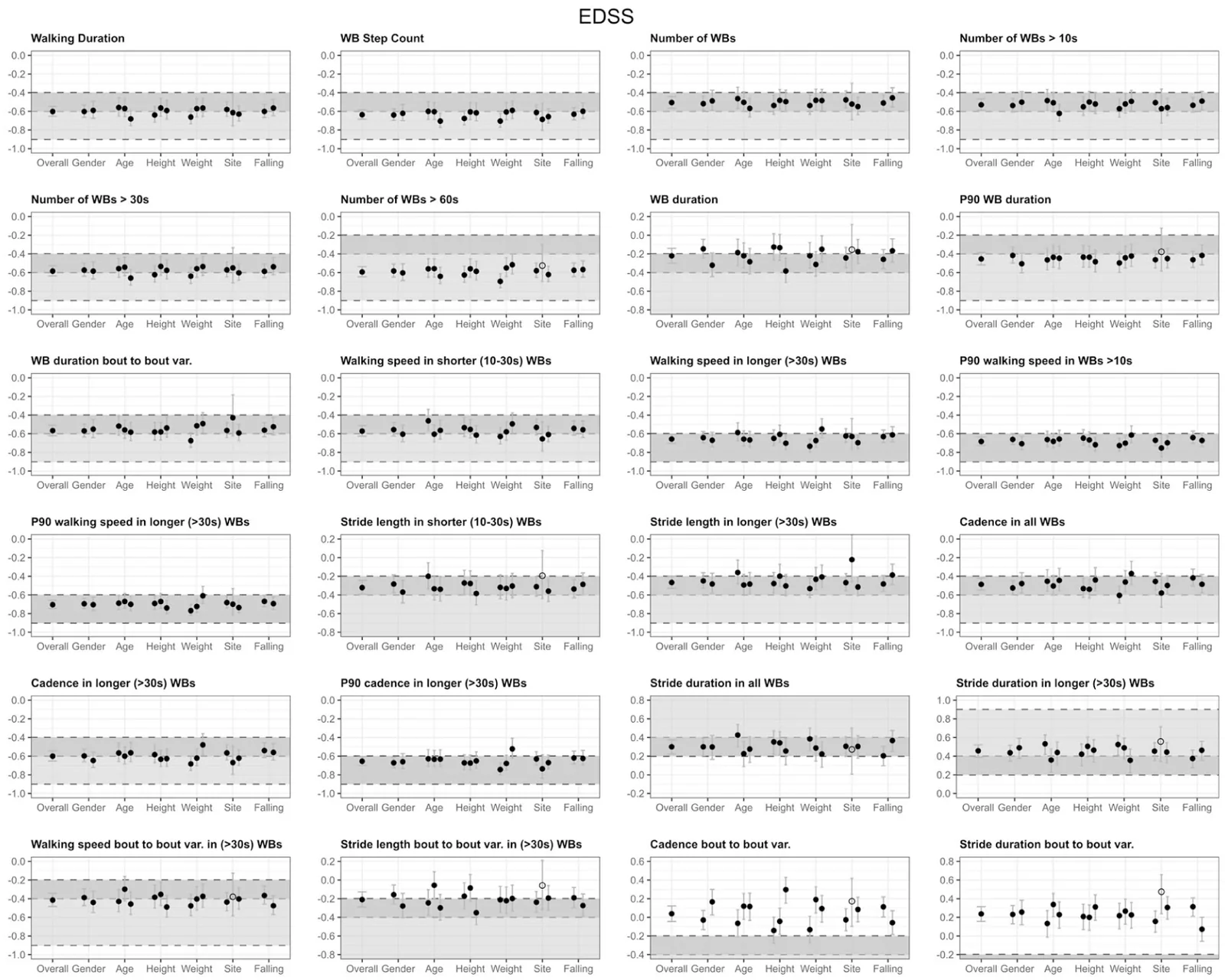
Correlation of each DMO with EDSS across different groups: gender (men and women), age (tertiles: <50, 50–57, and >57 years), height (tertiles: <165, 165–174, and >174 cm), weight (tertiles: <66, 66–88, and >88 kg), site (Germany, Italy, and England), and fall status (0 falls and ⩾1 fall). The dark grey areas represent the expected correlation ranges, while the light grey areas show extended acceptable ranges. Black dots indicate the observed correlations of these strata, while white dots indicate an insufficient sample size to detect the expected correlation. Where they fall within the acceptable limits, they support the validity of this DMO. EDSS: Expanded Disability Status Scale, P90: 90th percentile, var: variability.

**Figure 2. fig2-13524585261464537:**
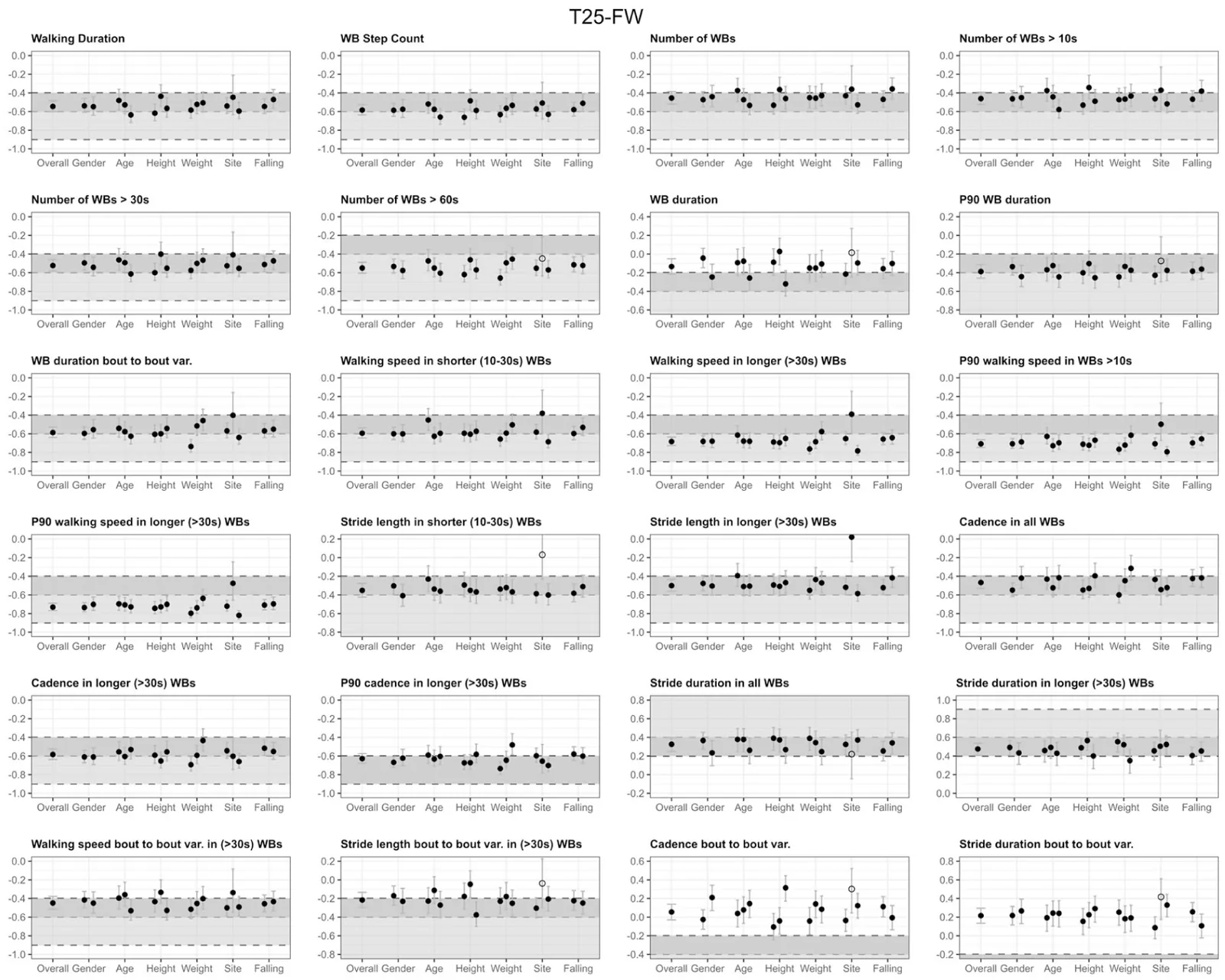
Correlation of each DMO with T25FW across different groups: gender (men and women), age (tertiles: <50, 50–57, and >57 years), height (tertiles: <165, 165–174, and >174 cm), weight (tertiles: <66, 66–88, and >88 kg), site (Germany, Italy, and England), and falling (0 falls and ⩾1 fall). The dark grey areas represent the expected correlation ranges, while the light grey areas show extended acceptable ranges. Black dots indicate the observed correlations of these strata, while white dots indicate an insufficient sample size to detect the expected correlation. Where they fall within the acceptable limits, they support the validity of this DMO. T25FW: Timed 25-Foot Walk, P90: 90th percentile, var: variability.

**Figure 3. fig3-13524585261464537:**
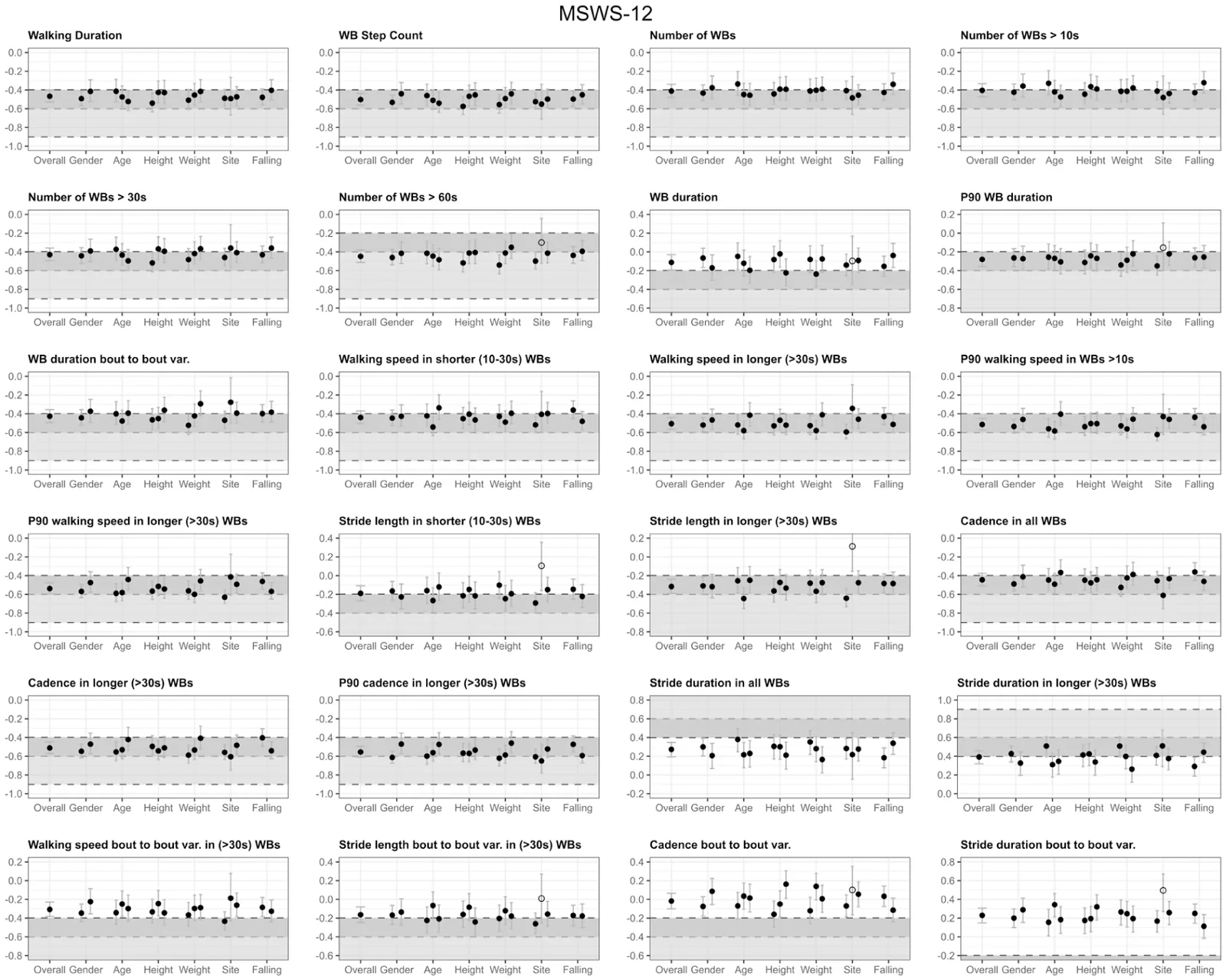
Correlation of each DMO with MSWS-12 across different groups: gender (men and women), age (tertiles: <50, 50–57, and >57 years), height (tertiles: <165, 165–174, and >174 cm), weight (tertiles: <66, 66–88, and >88 kg), site (Germany, Italy, and England), and falling (0 falls and ⩾1 fall). The dark grey areas represent the expected correlation ranges, while the light grey areas show extended acceptable ranges. Black dots indicate the observed correlations of these strata, while white dots indicate an insufficient sample size to detect the expected correlation. Where they fall within the acceptable limits, they support the validity of this DMO. MSWS-12: 12-item Multiple Sclerosis Walking Scale, P90: 90th percentile, var: variability.

**Figure 4.**
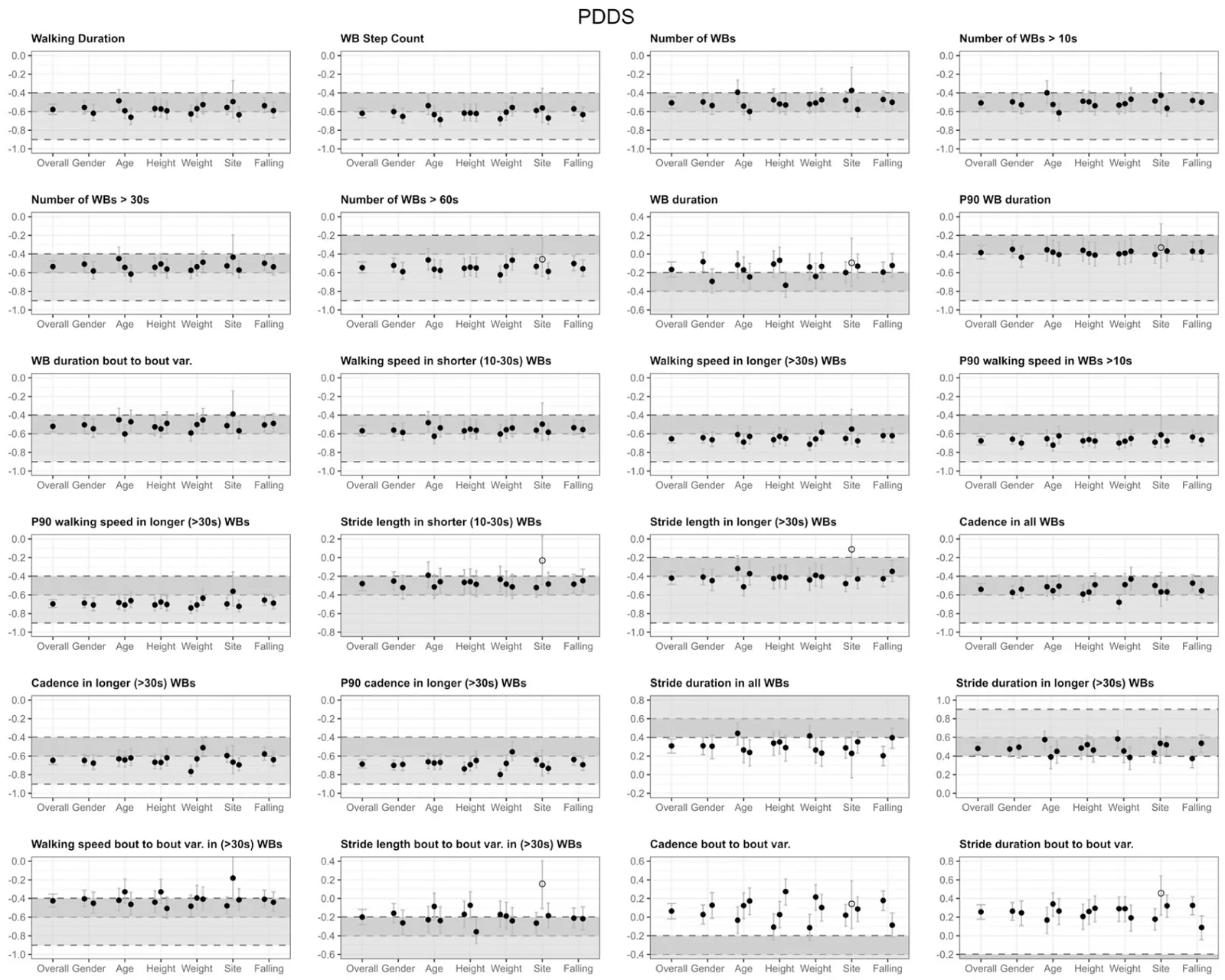
Correlation of each DMO with PDDS across different groups: gender (men and women), age (tertiles: <50, 50–57, and >57 years), height (tertiles: <165, 165–174, and >174 cm), weight (tertiles: <66, 66–88, and >88 kg), site (Germany, Italy, and England), and falling (0 falls and ⩾1 fall). The dark grey area represents the expected correlation range, while the light grey shows an extended acceptable range. Black dots indicate the observed correlations of these strata, while white dots indicate an insufficient sample size to detect the expected correlation. Where they fall within the acceptable limits, they support the validity of this DMO. PDDS: Patient-Determined Disease Steps, P90: 90th percentile, var: variability.

### Divergent validity

Negligible to weak coefficients were established for correlations between all 24 DMOs and systolic blood pressure (*r*/ ρ =|0.01|–|0.11|), consistent with our expectations (Table 2).

### Known-groups validity

Twenty-three DMOs (all except for cadence bout-to-bout variability) established significant differences between the three disease severity (EDSS) groups (see Figure 5).

**Figure 5.**
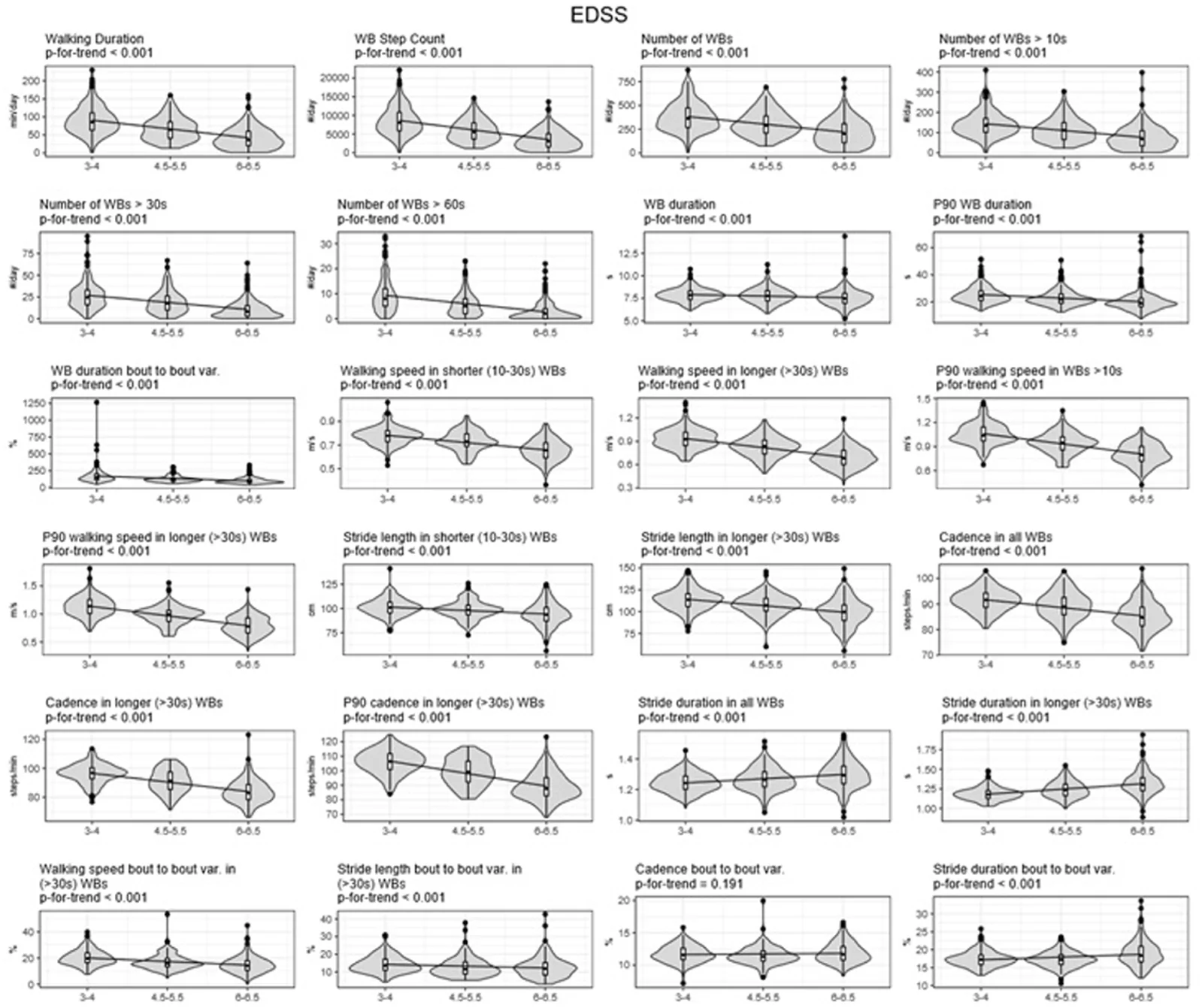
Distribution of DMO values and *p*-for-trend across EDSS severity groups. Violin plots show mean DMO values for EDSS groups (3–4, 4.5–5.5, and 6–6.5). *p* values represent linear trends from regression models; a significant *p*-for-trend (<0.05) supports known-groups validity. EDSS: Expanded Disability Status Scale, P90: 90th percentile, var: variability.

### Establishing construct validity

There was broad agreement that construct validity was met for 19 DMOs (Table 3), with the exceptions being WB duration, stride duration in all bouts, and all variability DMOs aside from walking speed bout-to-bout variability in more prolonged bouts.

**Table 3.** Expert consensus decisions about each DMO meeting construct validity or not.

DMO	Individual evaluation results	Consensus discussion	Consensus decision: meeting construct validity
**Walking activity**			
***Amount***			
Walking duration	100% in favour		**Yes**
WB step count	100% in favour		**Yes**
***Pattern***			
Number of WBs	100% in favour		**Yes**
Number of WBs >10 seconds	100% in favour		**Yes**
Number of WBs >30 seconds	100% in favour		**Yes**
Number of WBs >60 seconds	100% in favour		**Yes**
WB duration	0% in favour	• Weak correlations with EDSS only, with lower than expected correlations with the other clinical outcomes	**No**
P90 WB duration	100% in favour		**Yes**
WB duration bout-to-bout variability	100% in favour	• Construct validity was confirmed, but the expert group expressed caution in its interpretation, as the clinical meaning and relevance of this DMO remain unclear	**Yes**
**Gait**			
*Pace*			
Walking speed in shorter (10–30 seconds) WBs	100% in favour		**Yes**
Walking speed in longer (>30 seconds) WBs	100% in favour		**Yes**
P90 walking speed in WBs >10 seconds	100% in favour		**Yes**
P90 walking speed in longer (>30 seconds) WBs	100% in favour		**Yes**
Stride length in shorter (10–30 seconds) WBs	100% in favour	• Clinical expertise suggests caution in the use as measure of MS gait; stride length in short bouts likely to represent small spaces in the environment rather than disease process	**Yes**
Stride length in longer (>30 seconds) WBs	100% in favour	• Longer bouts may reflect outdoor walking and less of an environmental impact, as observed in short bouts	**Yes**
***Rhythm***			
Cadence in all WBs	100% in favour		**Yes**
Cadence in longer (>30 seconds) WBs	100% in favour		**Yes**
P90 cadence in longer (>30 seconds) WBs	100% in favour		**Yes**
Stride duration in all WBs	36% in favour	• Weak correlations exist for EDSS and T25FW, but expected correlations were not reached for MSWS-12 or PDDS. • Clinical expertise suggests caution in the use as measure of MS gait; stride duration in all WBs is likely to represent small spaces in the environment rather than disease process. • Joint decision to reject the DMO after discussion.	**No**
Stride duration in longer (>30 seconds) WBs	80% in favour	• Longer bouts may reflect outdoor walking and less of an environmental impact than observed in short bouts.	**Yes**
Bout-to-bout variability			
Walking speed bout-to-bout variability in longer (>30 seconds) WBs	91% in favour		**Yes**
Stride length bout-to-bout variability in longer (>30 seconds) WBs	45% in favour	• Expected correlations not met for MSWS-12 or PDDS. • No prior results in other studies for comparison. Following discussion, a joint decision that this DMO requires further exploration before use in pwMS.	**NO**
Cadence bout-to-bout variability	9% in favour	• No expected correlations were reached. • Does not discriminate between known (EDSS) groups. • No prior results in other studies for comparison. Following discussion, joint decision that this DMO requires further exploration before use in pwMS.	**No**
Stride duration bout-to-bout variability	27% in favour	• No expected correlations reached. • No prior results in other studies for comparison. Following the discussion, a joint decision was made that this DMO requires further exploration before use in pwMS.	**No**

EDSS: Expanded Disability Status Scale; MS type: multiple sclerosis type; MSWS-12: 12-item Multiple Sclerosis Walking Scale; T25FW: Timed 25-Foot Walk; PDDS: Patient-Determined Disease Steps; P90: 90th percentile.

## Discussion

This is the first multicentre study to systematically examine the construct validity of real-world DMOs in pwMS. Through the evaluation of convergent, divergent, and known-groups validity, our analysis shows that 19 out of 24 tested DMOs support construct validity, that is, all DMOs assessing walking activity (amount and pattern), except WB duration, and within the gait domains, all pace DMOs and all rhythm DMOs, except stride duration in all bouts, and walking speed bout-to-bout variability in longer bouts but no other measures of variability. Therefore, in pwMS, these DMOs appear to capture specific mobility aspects as intended, including clinical disability, walking capacity, perceived walking ability, and self-reported disability level.

### Walking activity DMOs

#### Amount

Step count has previously been assessed with methods of capture varying from pedometer to wrist- or lumbar-worn inertial sensor. It is apparent that in free-living conditions, pwMS with increasing severity demonstrate reductions in real-world activity and walking duration.^35,36^ Real-world step counts are also associated positively with gait speed and negatively with disease severity,^37^ self-reported disability,^38,39^ disease duration,^36^ and cortical grey matter and cervical spinal cord grey matter atrophy as observed on magnetic resonance imaging.^40^ Our results and expert consensus confirm the construct validity of these walking activity DMOs.

#### Pattern

Pattern DMOs have not been validated against other constructs in pwMS when WBs are separated by duration (overall, longer than 10, 30, and 60 seconds). Correlations with magnitudes close to expected were demonstrated between the constructs and DMOs, which is in line with the limited previous literature. Shema-Shiratzky et al.^36^ reported a correlation between the number of WBs >30 seconds and disease severity and real-world gait speed. In this study, pattern DMOs reflecting frequency of WBs are strongly related to the degree of disability, with frequency of WBs longer than 60 seconds being diminished across the cohort compared with lower duration WBs, but particularly low in more disabled pwMS (EDSS 6.0–6.5).

Meanwhile, WB duration did not meet construct validity as it does not appear to reflect the assessed constructs well, and the experts stipulated that the inclusion of many short WBs in this DMO likely reflects living environment, that is, small living spaces, rather than disease status. In contrast, P90 walking bout duration, which would also include outdoor walking, is negatively related to constructs of mobility disability in pwMS, in the range of expected magnitude, and is, therefore, considered a valid DMO. Furthermore, WB duration bout-to-bout variability, a surrogate for the ability to perform a variety of WBs, exhibited moderate negative correlations across constructs, as expected, and distinguished between groups, and consensus opinion supported this DMO. Previous studies have reported day-to-day variability of step counts and maximum walking distance in the real world^41–43^ but to the best of our knowledge, this is the first report of WB duration bout-to-bout variability.

### Gait DMOs

#### Pace

There is limited evidence on walking speed in pwMS in real-world conditions. A study on the effect of Fampridine on unsupervised walking using the actibelt® accelerometer in 28 pwMS with gait impairment (EDSS range: 4–6.5) indicated that mean daily walking speed had a moderate correlation with locomotor capacity (T25FW, 6MWT) and perceived impact of walking difficulty (MSWS-12).^44^ A further small cohort of people with mild MS (EDSS 1.0–2.5, *n* = 6) had a faster mean real-world walking speed compared with more moderately affected counterparts (EDSS 3.0–5.0, *n* = 5) with only a marginal difference in maximum walking speed.^37^ In this study, moderate correlations between all pace DMOs and mobility disability-related constructs were observed, consistent with expectations.

Walking speed for shorter and longer bouts, maximum walking speed, and stride length in both shorter and longer bouts have been found to support construct validity in pwMS. To our knowledge, this is the first time these variables have been assessed in this way. Regarding the clinical interpretation of stride length in shorter bouts, expert consensus suggests that caution should be noted regarding the clinical interpretation of DMOs in shorter bouts, as they may predominantly reflect environmental factors, particularly small indoor spaces, rather than disease status.

#### Rhythm

Prior literature indicates that real-world cadence may be used as an indicator of disability level.^36,45^ For stride duration, another prior study of real-world walking found a significant difference between EDSS 5.0–5.5 and 6.0–6.5 in short WBs (<50 steps), with stride time in longer WBs (51–100 steps) being reduced across both subgroups.^46^ In our analysis, cadence-related DMOs showed moderate correlations with related constructs, consistent with expectations, and were all considered valid constructs. In contrast, DMOs for stride duration produced weak correlations, and, across all WBs combined, stride duration was considered not to meet construct validity due to the absence of expected correlations with the MSWS-12 and PDDS. Stride duration in longer WBs was considered to meet construct validity, owing to moderate correlations with all constructs except MSWS-12. This difference in construct validity between stride DMOs was hypothesised to better reflect outdoor walking and, therefore, to be less constrained by environmental effects than short bouts.

#### Bout-to-bout variability

Fluctuations in gait performance between WBs have not previously been researched in pwMS, and expert consensus suggested that, aside from walking speed bout-to-bout variability in long bouts, all bout-to-bout variability DMOs require further evaluation. Walking speed bout-to-bout variability produced moderate correlations with constructs, and the DMO reflects the slower pace observed in people with advanced disability.

### Strengths and limitations

The broad inclusion criteria of this study, which encompassed any person with MS experiencing subjective or objective worsening, together with the multicentre design and data collection spanning multiple seasons, support the validity of our results to the broader MS population. The analytical methods, rigorous data cleaning, and use of a priori hypotheses informed by expert consensus further strengthen the validity of the results. While we included multiple widely used and complementary clinical anchors, some dimensions of mobility relevant to pwMS, such as contextual or compensatory behaviours, were not measured; further research should explore whether digital mobility outcomes (DMOs) reflect them.

In addition, this study evaluated construct validity only. Other important clinicometric measurement properties, including the ability to detect change and the minimally important difference, are being assessed in ongoing longitudinal analyses within the overarching project.

### Implications

These findings support further evaluation and potential use in clinical research settings for DMOs as indicators of mobility performance in pwMS. The observed associations with established clinical measures suggest that DMOs may capture clinically meaningful aspects of walking performance. This has important implications for their use in clinical trials, particularly for detecting subtle or early changes in mobility and for remote monitoring in routine care. However, it is our opinion that DMOs should be interpreted as complementary rather than replacement measures, and their full integration into clinical decision-making requires further longitudinal validation.

## Conclusion

This comprehensive construct validity analysis represents a significant step forward in using digital tools to assess mobility in pwMS. A total of 19 walking activity and gait DMOs covering domains of amount, pattern, pace, rhythm, and bout-to-bout variability are shown to be objective and valid outcomes in MS. The findings suggest that DMOs may capture clinically relevant aspects of real-world walking behaviour that may not be fully reflected by conventional assessments. While further longitudinal work is needed to establish responsiveness and prognostic value, these results contribute to the growing evidence base supporting the use of DMOs in MS research and, potentially, in future models of remote monitoring and care.

## Supplemental Material

sj-docx-1-msj-10.1177_13524585261464537 – Supplemental material for Testing the construct validity of real-world digital mobility outcomes in multiple sclerosis Supplemental material, sj-docx-1-msj-10.1177_13524585261464537 for Testing the construct validity of real-world digital mobility outcomes in multiple sclerosis by Gavin Brittain, Michael Long, Ellen Buckley, Clemens Becker, Joren Buekers, Martí de las Heras, Claudia Armengol, Sarah Koch, Julia Braun, Brian Caufield, Giancarlo Comi, Gloria Dalla Costa, Anja Frei, Heiko Gassner, Anisoara Ionescu, Letizia Leocani, Lynn Rochester, Veit Rothhammer, David Singleton, Klarissa Hanja Stuerner, David Paling, Krishnan Padmakumari Sivaraman Nair, Arshad Majid, Giampaolo Brichetto, Beatrix Vereijken, Vita Lanfranchi, Carl-Philipp Jansen, Lou Sutcliffe, Silvia Del Din, Isabel Neatrour, Andrea Cereatti, Walter Maetzler, Clint Hansen, Daniel Rooks, Basil Sharrack and Judith Garcia-Aymerich in Multiple Sclerosis Journal

## Data Availability

All data will be made public in due course. See www.mobilise-d.eu for the availability timeline.
