# Supplementary material for: Testing the construct validity of real-world digital mobility outcomes in multiple sclerosis
Source: Mult Scler. 2026 Aug 2;32(13):1431–46. doi: 10.1177/13524585261464537 (PMC13748489; doi:10.1177/13524585261464537)
Supplement: Supplementary file 1 [file sj-docx-1-msj-10.1177_13524585261464537.docx]

**Table S1: Description of digital mobility outcomes used**

|  | **Weekly calculation** |
| --- | --- |
| **Walking activity** |  |
| *Amount* |  |
| Walking Duration (min/day) | Weekly mean of daily sum of WB duration using all WBs |
| WB Step Count (steps/day) | Weekly mean of daily sum of steps using all WBs |
| *Pattern* |  |
| Number of WBs (n) | Weekly mean of daily sum of all WBs |
| Number of WBs > 10s (n) | Weekly mean of daily sum of number of WBs longer than 10s |
| Number of WBs > 30s (n) | Weekly mean of daily sum of number of WBs longer than 30s |
| Number of WBs > 60s (n) | Weekly mean of daily sum of number of WBs longer than 60s |
| WB duration (s) | Weekly mean of daily median of WB duration using all WBs |
| P90 WB duration (s) | Weekly mean of daily P90 of WB duration using all WBs |
| WB duration bout to bout variability (%) | Weekly mean of daily COV of WB duration using all WBs |
| **Gait** |  |
| *Pace* |  |
| Walking speed in shorter (10-30s) WBs (m/s) | Weekly mean of daily mean walking speed using WBs between 10s and 30s |
| Walking speed in longer (>30s) WBs (m/s) | Weekly mean of daily mean walking speed using WBs longer than 30s |
| P90 walking speed in WBs >10s (m/s) | Weekly mean of daily P90 of walking speed using WBs longer than 10s |
| P90 walking speed in longer (>30s) WBs (m/s) | Weekly mean of daily P90 of walking speed using WBs longer than 30s |
| Stride length in shorter (10-30s) WBs (m) | Weekly mean of daily mean of stride length using WBs between 10s and 30s |
| Stride length in longer (>30s) WBs (m) | Weekly mean of daily mean of stride length using WBs longer than 30s |
| *Rhythm* |  |
| Cadence in all WBs (steps/min) | Weekly mean of daily mean of cadence using all WBs |
| Cadence in longer (>30s) WBs (step/min) | Weekly mean of daily mean of cadence using WBs longer than 30s |
| P90 cadence in longer (>30s) WBs (steps/min) | Weekly mean of daily P90 using WBs longer than 30s |
| Stride duration in all WBs (s) | Weekly mean of daily mean of stride duration using all WBs |
| Stride duration in longer (>30s) WBs (s) | Weekly mean of daily mean of stride duration using WBs longer than 30s |
| *Bout-to-bout variability* |  |
| Walking speed bout to bout variability in longer (>30s) WBs (%) | Weekly mean of daily coefficient of variation of walking speed using WBs longer than 30s |
| Stride length bout to bout variability in longer (>30s) WBs (%) | Weekly mean of daily coefficient of variation of stride length using WBs longer than 30s |
| Cadence bout to bout variability (%) | Weekly mean of daily coefficient of variation using all WBs |
| Stride duration bout to bout variability (%) | Weekly mean of daily coefficient of variation of stride duration using all WBs |

**Table S2:** Expected correlation coefficient ranges between DMOs and related constructs derived from previous research, pilot testing and expert consultation^1-19^

|  | EDSS | T25FW | MSWS-12 | PDDS |
| --- | --- | --- | --- | --- |
| **Walking activity** |  |  |  |  |
| *Amount* |  |  |  |  |
| Walking Duration (min/day) | -0.4 to -0.6 | -0.4 to -0.6 | -0.4 to -0.6 | -0.4 to -0.6 |
| WB Step Count (steps/day) | -0.4 to -0.6 | -0.4 to -0.6 | -0.4 to -0.6 | -0.4 to -0.6 |
| *Pattern* |  |  |  |  |
| Number of WBs (n) | -0.4 to -0.6 | -0.4 to -0.6 | -0.4 to -0.6 | -0.4 to -0.6 |
| Number of WBs > 10s (n) | -0.4 to -0.6 | -0.4 to -0.6 | -0.4 to -0.6 | -0.4 to -0.6 |
| Number of WBs > 30s (n) | -0.4 to -0.6 | -0.4 to -0.6 | -0.4 to -0.6 | -0.4 to -0.6 |
| Number of WBs > 60s (n) | -0.2 to -0.4 | -0.2 to -0.4 | -0.2 to -0.4 | -0.2 to -0.4 |
| WB duration (s) | -0.2 to -0.4 | -0.2 to -0.4 | -0.2 to -0.4 | -0.2 to -0.4 |
| P90 WB duration (s) | -0.2 to -0.4 | -0.2 to -0.4 | -0.2 to -0.4 | -0.2 to -0.4 |
| WB duration bout to bout variability (%) | -0.4 to -0.6 | -0.4 to -0.6 | -0.4 to -0.6 | -0.4 to -0.6 |
| **Gait** |  |  |  |  |
| *Pace* |  |  |  |  |
| Walking speed in shorter (10-30s) WBs (m/s) | -0.4 to -0.6 | -0.4 to -0.6 | -0.4 to -0.6 | -0.4 to -0.6 |
| Walking speed in longer (>30s) WBs (m/s) | -0.6 to -0.9 | -0.4 to -0.6 | -0.4 to -0.6 | -0.4 to -0.6 |
| P90 walking speed in WBs >10s (m/s) | -0.6 to -0.9 | -0.4 to -0.6 | -0.4 to -0.6 | -0.4 to -0.6 |
| P90 walking speed in longer (>30s) WBs (m/s) | -0.6 to -0.9 | -0.4 to -0.6 | -0.4 to -0.6 | -0.4 to -0.6 |
| Stride length in shorter (10-30s) WBs (m) | -0.2 to -0.4 | -0.2 to -0.4 | -0.2 to -0.4 | -0.2 to -0.4 |
| Stride length in longer (>30s) WBs (m) | -0.4 to -0.6 | -0.4 to -0.6 | -0.2 to -0.4 | -0.2 to -0.4 |
| *Rhythm* |  |  |  |  |
| Cadence in all WBs (steps/min) | -0.4 to -0.6 | -0.4 to -0.6 | -0.4 to -0.6 | -0.4 to -0.6 |
| Cadence in longer (>30s) WBs (step/min) | -0.4 to -0.6 | -0.4 to -0.6 | -0.4 to -0.6 | -0.4 to -0.6 |
| P90 cadence in longer (>30s) WBs (steps/min) | -0.6 to -0.9 | -0.6 to -0.9 | -0.4 to -0.6 | -0.4 to -0.6 |
| Stride duration in all WBs (s) | 0.2 to 0.4 | 0.2 to 0.4 | 0.4 to 0.6 | 0.4 to 0.6 |
| Stride duration in longer (>30s) WBs (s) | 0.2 to 0.4 | 0.4 to 0.6 | 0.4 to 0.6 | 0.4 to 0.6 |
| *Bout-to-bout variability* |  |  |  |  |
| Walking speed bout to bout variability in longer (>30s) WBs (%) | -0.2 to -0.4 | -0.4 to -0.6 | -0.4 to -0.6 | -0.4 to -0.6 |
| Stride length bout to bout variability in longer (>30s) WBs (%) | -0.2 to -0.4 | -0.2 to -0.4 | -0.2 to -0.4 | -0.2 to -0.4 |
| Cadence bout to bout variability (%) | -0.2 to -0.4 | -0.2 to -0.4 | -0.2 to -0.4 | -0.2 to -0.4 |
| Stride duration bout to bout variability (%) | -0.2 to -0.4 | -0.2 to -0.4 | -0.2 to -0.4 | -0.2 to -0.4 |

EDSS: Expanded Disability Status Scale; MSWS-12: 12-item Multiple Sclerosis Walking Scale; T25FW: Timed 25-Foot Walk; PDDS: Patient Determined Disease Steps, P90: 90^th^ percentile.

References for table S1:

1.	Shema-Shiratzky S, Hillel I, Mirelman A, et al. A wearable sensor identifies alterations in community ambulation in multiple sclerosis: contributors to real-world gait quality and physical activity. Journal of Neurology 2020; 267: 1912–1921. DOI: 10.1007/s00415-020-09759-7.

2.	Zanotto T, Galperin I, Mirelman A, et al. Association Between Frailty and Free-Living Walking Performance in People With Multiple Sclerosis. Physical Therapy 2023; 103. DOI: 10.1093/ptj/pzad032.

3.	Motl RW, Dlugonski D, Suh Y, et al. Accelerometry and its association with objective markers of walking limitations in ambulatory adults with multiple sclerosis. Arch Phys Med Rehabil 2010; 91: 1942–1947. DOI: 10.1016/j.apmr.2010.08.011.

4.	Motl RW, McAuley E, Snook EM, et al. Accuracy of two electronic pedometers for measuring steps taken under controlled conditions among ambulatory individuals with multiple sclerosis. Mult Scler 2005; 11: 343–345. DOI: 10.1191/1352458505ms1161oa.

5.	Motl RW, Pilutti L, Sandroff BM, et al. Accelerometry as a measure of walking behavior in multiple sclerosis. Acta Neurologica Scandinavica 2013; 127: 384–390. DOI: https://doi.org/10.1111/ane.12036.

6.	Motl RW, Pilutti LA, Learmonth YC, et al. Clinical Importance of Steps Taken per Day among Persons with Multiple Sclerosis. PLOS ONE 2013; 8: e73247. DOI: 10.1371/journal.pone.0073247.

7.	Weikert M, Motl RW, Suh Y, et al. Accelerometry in persons with multiple sclerosis: Measurement of physical activity or walking mobility? Journal of the Neurological Sciences 2010; 290: 6–11. DOI: 10.1016/j.jns.2009.12.021.

8.	Zheng P, Jeng B, Huynh TLT, et al. Free-Living Peak Cadence in Multiple Sclerosis: A New Measure of Real-World Walking? Neurorehabilitation and Neural Repair 2023; 37: 716–726. DOI: 10.1177/15459683231206741.

9.	Zheng P, Sandroff BM and Motl RW. Free-living ambulatory physical activity and cognitive function in multiple sclerosis: the significance of step rate vs. step volume. Journal of Neurology 2024; 271: 1638–1648. DOI: 10.1007/s00415-023-12169-0.

10.	Block VJ, Bove R, Zhao C, et al. Association of Continuous Assessment of Step Count by Remote Monitoring With Disability Progression Among Adults With Multiple Sclerosis. JAMA Netw Open 2019; 2: e190570. 20190301. DOI: 10.1001/jamanetworkopen.2019.0570.

11.	Block VJ, Lizée A, Crabtree-Hartman E, et al. Continuous daily assessment of multiple sclerosis disability using remote step count monitoring. J Neurol 2017; 264: 316–326. 20161128. DOI: 10.1007/s00415-016-8334-6.

12.	Block VJ, Waliman M, Xie Z, et al. Making Every Step Count: Minute-by-Minute Characterization of Step Counts Augments Remote Activity Monitoring in People With Multiple Sclerosis. Front Neurol 2022; 13: 860008. 20220523. DOI: 10.3389/fneur.2022.860008.

13.	Block VJ, Zhao C, Hollenbach JA, et al. Validation of a consumer-grade activity monitor for continuous daily activity monitoring in individuals with multiple sclerosis. Mult Scler J Exp Transl Clin 2019; 5: 2055217319888660. 20191121. DOI: 10.1177/2055217319888660.

14.	Neven A, Vanderstraeten A, Janssens D, et al. Understanding walking activity in multiple sclerosis: step count, walking intensity and uninterrupted walking activity duration related to degree of disability. Neurol Sci 2016; 37: 1483–1490. 20160520. DOI: 10.1007/s10072-016-2609-7.

15.	Dastagir A, Healy BC, Chua AS, et al. Brain and spinal cord MRI lesions in primary progressive vs. relapsing-remitting multiple sclerosis. eNeurologicalSci 2018; 12: 42–46. DOI: https://doi.org/10.1016/j.ensci.2018.07.002.

16.	Albrecht H, Wötzel C, Erasmus LP, et al. Day-to-day variability of maximum walking distance in MS patients can mislead to relevant changes in the Expanded Disability Status Scale (EDSS): average walking speed is a more constant parameter. Mult Scler 2001; 7: 105–109. 2001/06/27. DOI: 10.1177/135245850100700206.

17.	Cavanaugh JT, Gappmaier VO, Dibble LE, et al. Ambulatory activity in individuals with multiple sclerosis. J Neurol Phys Ther 2011; 35: 26–33. DOI: 10.1097/NPT.0b013e3182097190.

18.	Storm FA, Nair KPS, Clarke AJ, et al. Free-living and laboratory gait characteristics in patients with multiple sclerosis. PLOS ONE 2018; 13: e0196463. DOI: 10.1371/journal.pone.0196463.

19.	Micó-Amigo ME, Bonci T, Paraschiv-Ionescu A, et al. Assessing real-world gait with digital technology? Validation, insights and recommendations from the Mobilise-D consortium. Journal of NeuroEngineering and Rehabilitation 2023; 20: 78. DOI: 10.1186/s12984-023-01198-5.

|  | **Excluded n = 46  (8%)** | **Included n = 556 (92%)** | **p-value** |
| --- | --- | --- | --- |
| Age, mean (SD) | 53 (12) | 52 (11) | 0.453 |
| Gender, female, n (%) | 27 (59%) | 360 (65%) | 0.421 |
| Marital status, married, n (%) | 30 (65%) | 375 (67%) | 0.310 |
| Education, n (%) |  |  | 0.606 |
| *College Degree or higher* | 12 (26%) | 190 (34%) |  |
| *Tertiary education* | 14 (30%) | 152 (27%) |  |
| *High school* | 16 (35%) | 143 (26%) |  |
| *Less than high school (<10 yrs)/ others* | 4 (9%) | 71 (13%) |  |
| Site, n (%) |  |  | 0.140 |
| *Kiel* | 1 (2%) | 44 (8%) |  |
| *Erlangen* | 3 (7%) | 14 (3%) |  |
| *Sheffield* | 20 (43%) | 280 (50%) |  |
| *Milan* | 22 (48%) | 218 (39%) |  |
| Self-reported diagnosis of comorbidities, n (%) |  |  | - |
| *Arthritis* | 6 (13%) | 62 (11%) |  |
| *Osteoporosis* | 3 (7%) | 44 (8%) |  |
| *Chronic heart failure* | 2 (4%) | 7 (1%) |  |
| *Neurological disease* | 46 (100%) | 555 (100%) |  |
| *Diabetes* | 2 (4%) | 20 (4%) |  |
| *Depression* | 14 (30%) | 121 (22%) |  |
| *Anxiety* | 6 (13%) | 75 (13%) |  |
| *Visual Impairment* | 7 (15%) | 69 (12%) |  |
| *Hearing Impairment* | 1 (2%) | 40 (7%) |  |
| *Obesity* | 8 (17%) | 124 (22%) |  |
| MS Type, n (%) |  |  | 0.081 |
| *Primary Progressive* | 4 (9%) | 59 (11%) |  |
| *Secondary Progressive* | 24 (52%) | 195 (35%) |  |
| *Relapsing Remitting* | 18 (39%) | 300 (54%) |  |
| MSWS-12, med (Q1-Q3) | 44 (30 - 51) | 41 (28 - 51) | 0.207 |
| T25FW (s), med (Q1-Q3) | 9 (7 - 14) | 8 (6 - 11) | 0.033 |
| PDDS (s), med (Q1-Q3) | 4 (3 - 5) | 3 (2 - 5) | 0.169 |
| Systolic blood pressure (mmHg), mean (SD) | 129 (19) | 130 (18) | 0.611 |
| Walking Aids, n (%) |  |  |  |
| Indoor | 16 (35%) | 136 (24%) | 0.160 |
| Outdoor | 31 (67%) | 325 (58%) | 0.274 |
| Falls in previous 12 months, median (Q1-Q3) | 2 (0 - 6) | 1 (0 - 3) | 0.324 |
| *0 falls* | 19 (41%) | 234 (42%) |  |
| *1 fall* | 4 (9%) | 68 (12%) |  |
| *>=2 falls* | 23 (50%) | 254 (46%) |  |
| EDSS Categories, n (%) |  |  | 0.194 |
| *3.0-4.0* | 12 (26%) | 192 (35%) |  |
| *4.5-5.5* | 7 (15%) | 120 (22%) |  |
| *6.0-6.5* | 26 (57%) | 241 (43%) |  |

**Table S3:** Statistical comparison between included and excluded participants. Of the 46 (8%) excluded participants, 30 (5%) had missing data and 16 (2.7%) did not meet the minimum wear time.

EDSS: Expanded Disability Status Scale; MS type: Multiple Sclerosis type; MSWS-12: 12-item Multiple Sclerosis Walking Scale; T25FW: Timed 25-Foot Walk; PDDS: Patient Determined Disease Steps
